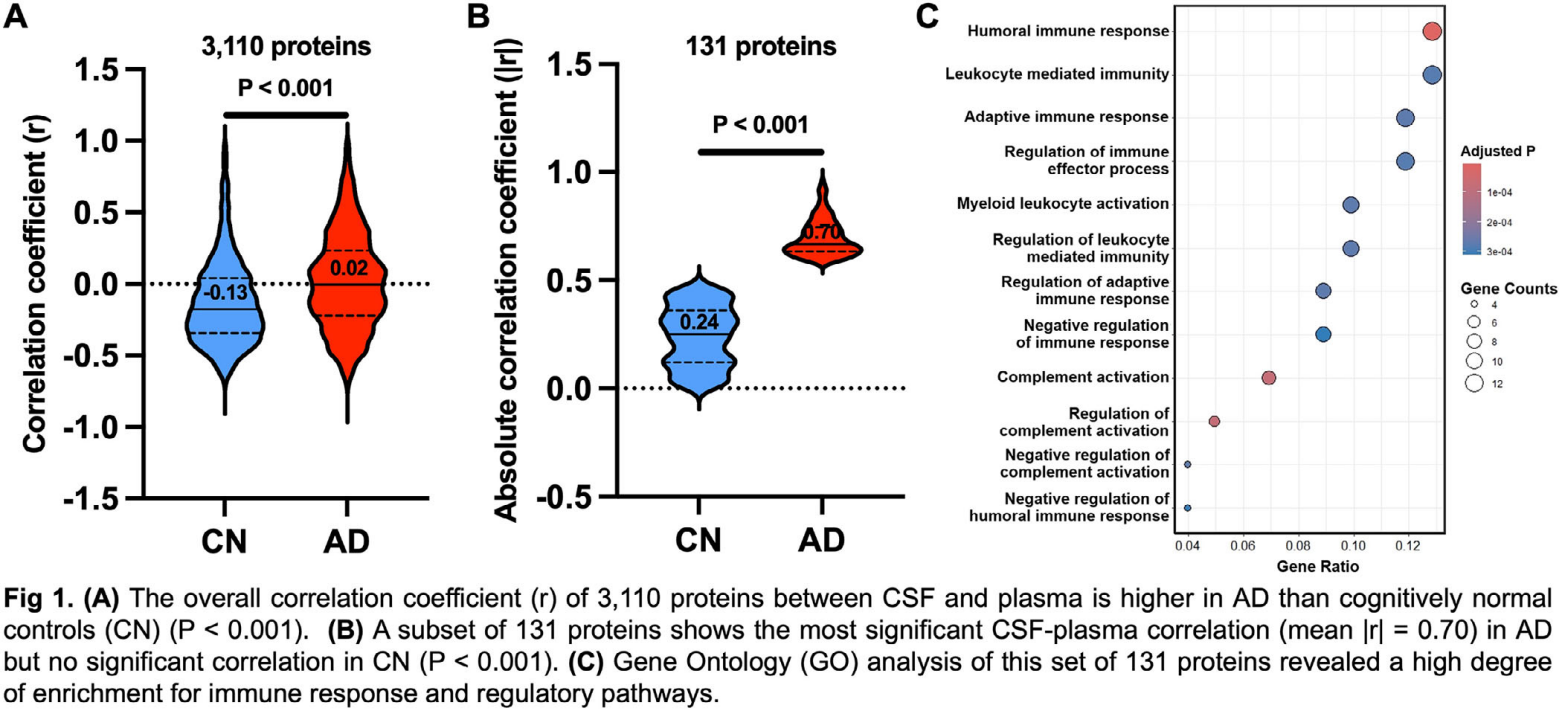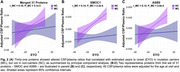# Coordination of CSF and Peripheral Circulation Proteomics across the AD Spectrum

**DOI:** 10.1002/alz70861_108628

**Published:** 2025-12-23

**Authors:** Jijing Wang, Stephanie A. Schultz, Erik C.B. Johnson, Eric B. Dammer, Lei Liu, Celeste M. Karch, Eric McDade, Carlos Cruchaga, Hyun‐Sik Yang, Jasmeer P. Chhatwal, the Dominantly Inherited Alzheimer Network (DIAN)

**Affiliations:** ^1^ Mass General Brigham, Boston, MA USA; ^2^ Harvard Medical School, Boston, MA USA; ^3^ The Broad Institute of MIT and Harvard, Cambridge, MA USA; ^4^ Goizueta Alzheimer's Disease Research Center, Emory University, Atlanta, GA USA; ^5^ Washington University School of Medicine, St. Louis, MO USA

## Abstract

**Background:**

Progressive disruption of the blood–brain barrier (BBB) is believed to contribute to Alzheimer’s disease (AD) pathophysiology. BBB deterioration may increase crosstalk between the central nervous system (CNS) and the periphery, potentially resulting in more closely aligned inflammatory states across compartments. Using proteomic profiling of paired blood and cerebrospinal fluid (CSF) samples, we investigated CNS and peripheral protein coordination in late‐onset AD (LOAD) and dominantly inherited AD.

**Method:**

We analyzed data from 36 participants (18 LOAD, 18 controls) in the Emory 3 Platform study, along with 132 mutation carriers (MC) and 94 non‐carriers (NC) from the Dominantly Inherited Alzheimer Network (DIAN). Paired CSF and plasma proteomic data were generated using the SomaLogic SomaScan 7K platform. In the Emory dataset, linear regression models assessed CSF–plasma protein associations in AD and controls, respectively. Gene Ontology (GO) overrepresentation analysis was performed on identified protein sets. In DIAN, estimated years to symptom onset (EYO) was calculated as the visit age minus the expected age of symptom onset (AAO). Associations between CSF/plasma protein ratios and EYO were analyzed using linear regression adjusted for age and sex.

**Result:**

In the Emory cohort, CSF‐plasma correlations across all 3,110 proteins were significantly higher in AD than in controls (Figure 1A). Of these, 131 proteins showed highly significant CSF–plasma correlations only in AD (Figure 1B). GO analysis of these proteins revealed strong enrichment of immune response and regulatory pathways (Figure 1C). In DIAN, CSF/plasma ratios for 31 proteins exhibited significant associations with EYO in MC compared to NC. These CSF/plasma ratios increased with EYO in MC compared to NC (Figure 2A), suggesting changes that are associated with AD progression. Two representative proteins from this set (SMOC1: Figure 2B, ASB9: Figure 2C) are shown for illustration purposes.

**Conclusion:**

We identified a set of proteins with altered CSF–plasma gradients in AD, likely reflecting BBB dysfunction and increased CNS–periphery immune interaction. These proteins may offer insights into BBB dysfunction in pre‐symptomatic and symptomatic AD, and potentially serve as biomarkers of BBB dynamics throughout disease course.